# A Review on the Gluten-Free Diet: Technological and Nutritional Challenges

**DOI:** 10.3390/nu10101410

**Published:** 2018-10-02

**Authors:** Dalia El Khoury, Skye Balfour-Ducharme, Iris J. Joye

**Affiliations:** 1 Department of Family Relations & Applied Nutrition, University of Guelph, 50 Stone Road East, Guelph, ON N1G 2W1, Canada; balfourd@uoguelph.ca; 2 Department of Food Science, University of Guelph, 50 Stone Road East, Guelph, ON N1G 2W1, Canada; ijoye@uoguelph.ca

**Keywords:** celiac disease, gluten, wheat, auto-immune disease, non-celiac gluten sensitivity, gluten-free, gluten-related disorders, weight management, nutritional adequacy, product development

## Abstract

Consumers, food manufacturers and health professionals are uniquely influenced by the growing popularity of the gluten-free diet. Consumer expectations have urged the food industry to continuously adjust and improve the formulations and processing techniques used in gluten-free product manufacturing. Health experts have been interested in the nutritional adequacy of the diet, as well as its effectiveness in managing gluten-related disorders and other conditions. In this review, we aim to provide a clear picture of the current motivations behind the use of gluten-free diets, as well as the technological and nutritional challenges of the diet as a whole. Alternative starches and flours, hydrocolloids, and fiber sources were found to play a complex role in mimicking the functional and sensory effects of gluten in gluten-free products. However, the quality of gluten-free alternatives is often still inferior to the gluten-containing products. Furthermore, the gluten-free diet has demonstrated benefits in managing some gluten-related disorders, though nutritional imbalances have been reported. As there is limited evidence supporting the use of the gluten-free diet beyond its role in managing gluten-related disorders, consumers are urged to be mindful of the sensorial limitations and nutritional inadequacies of the diet despite ongoing strategies to improve them.

## 1. Introduction

In cereal processing, gluten refers to the combined gliadin (prolamin) and glutenin (glutelin) fraction of wheat [1]. The gluten protein fraction displays unique structure building properties that are used in food processing. These structure building properties are also reflected in the terminology, as gluten is essentially the Latin translation of “glue” [2]. Gluten in wheat flour forms a three-dimensional protein network upon proper hydration and mixing. These network-forming properties are utilized in baking applications to create viscoelastic dough matrices. Besides network formation, gluten functionality in food includes water binding and viscosity yielding, which make gluten a widely used food additive [3].

Gluten is also a nutritional term used to refer to certain cereal prolamins, i.e., the ethanol-soluble proteins of wheat, rye, barley, their cross bred grains, and possibly oats [1,4]. These prolamins are very important in the context of coeliac disease (CD), non-coeliac gluten sensitivity (NCGS), gluten ataxia (GA) and dermatitis herpetiformis (DH). For people suffering from CD, NCGS, GA, and DH, the only effective “treatment” to date consists of eliminating gluten completely and life-long out of their diet. In what follows, general information on gluten-free products and the diet is provided. Challenges when formulating gluten-free products, consumers’ motivations, knowledge and attitudes, as well as the nutritional and therapeutic implications of the gluten-free diet, are also further elaborated on.

## 2. Gluten-Free Products

Global market data indicate that gluten-free product sales are forecasted to increase by a compound annual growth rate of 10.4% between 2015 to 2020 [5]. As the clinical application and popularity of the gluten-free diet escalate, consumer demands righteously continue to influence the food market and labelling standards of gluten-free products. In 2013, the European Union Regulation 609/2013 set out rules on compositional and labelling requirements for gluten-free products [6]. These guidelines ensure that people who are intolerant to gluten are adequately informed of the difference between foods that are naturally free of gluten and foods that are produced, prepared and/or processed in order to reduce their gluten content [6]. In the same year, the Food and Drug Administration ruled that products labelled “gluten-free” cannot surpass a threshold of 20 parts per million, although the official compliance date was set for 2014 [7]. This guideline helps gluten-wary customers to navigate the current market and protect themselves from consuming products that may exacerbate their symptoms and/or activate immune-mediated mucosal damage even in the absence of symptoms. The gluten-free diet encompasses food groups that are naturally devoid of gluten, such as fresh fruit, vegetables, seafood, meat, poultry, legumes, nuts, and most dairy products [8]. However, some of these products may also contain “hidden” gluten. Hence, product labels and ingredient lists need to be carefully reviewed. For the traditional gluten-containing foods, such as bakery products, there is currently a wide variety of gluten-free options available that use gluten-free cereals and pseudocereals, such as rice, corn, quinoa, millet, and amaranth as their base ingredients [9].

### 2.1. Gluten Functionality

The unique properties of wheat flour can primarily be ascribed to its gluten fraction. Gluten has unequalled network forming properties, which are important for products that are made with hard wheat varieties, and typically involve an intermediate cohesive dough stage during their production process [10]. Examples of such products are bread, pasta, and pretzels. Gliadin is the 70% ethanol-soluble protein fraction of wheat flour and is essentially present in wheat grain extracts as monomeric proteins [1]. Glutenin, on the other hand, is the protein fraction that cannot be extracted with water, diluted salt solutions, and 70% ethanol, and is often referred to as the polymeric gluten [1]. Both gliadin and glutenin are important for network formation and the quality of the final food product. Although the exact structure and interactions of this protein network are still under debate, it is widely accepted that gliadin has a viscosity-increasing effect, whilst the elastic properties of the network and wheat flour dough predominantly stem from the glutenin fraction [1,10]. For soft wheat products such as cakes and cookies, the gluten network-forming properties are not as crucial, but gluten is believed to nevertheless contribute to final product structure and texture [11,12]. In what follows, focus will be laid on gluten-free bread products as bread is an important staple food and its quality heavily relies on gluten properties and functionality. Hence, bread is one of the more challenging food products when making gluten-free alternatives.

### 2.2. Gluten Replacement Strategies

Bread baking without gluten essentially removes the most crucial ingredient for product structure and quality. This presents a major challenge to bakers and cereal researchers. In addition, gluten-free products are often consumed by people who have had the opportunity to try and enjoy gluten-containing foods. These consumers, therefore, already have product expectations in terms of texture, structure, flavor and overall quality imprinted. Besides designing gluten-free bread products in such way that they closely mimic the texture of gluten-containing bread products, they also need to have the same sensory profile and shelf life [13]. One possible strategy that has been identified for matching the volatile flavor of wheat-containing products is combining proline and glucose in the gluten-free product recipe, as these are precursors of the volatile components found in wheat-based bread products [14]. In what follows, different ingredient and processing strategies will be reviewed, with a focus on texture, structure, and volume of gluten-free bread products. Most of the research done on gluten-free bread formulations focused on the effect of an extra ingredient or a (partial) replacement of one of the base ingredients with a promising other compound. The product against which these new formulations have been tested in terms of quality is almost always the gluten-free product, which does not contain the extra ingredient or the replacement compound. However, as stated above, the real goal of formulating high quality gluten-free products is to achieve the same product characteristics and quality of a regular gluten-containing bread. Therefore, it would be more useful to compare the obtained gluten-free “dough” and bread characteristics with those of an actual similar gluten-containing system.

#### 2.2.1. Ingredients

Imitating the cohesiveness and elasticity of a gluten-containing dough was attempted using a wide range of alternative raw ingredients and/or additives. Gluten-replacing ingredients include starches, gluten-free flours of cereals/pseudocereals, hydrocolloids, and proteins. Minor ingredients that are added to help build and strengthen gluten-free dough and bread structure are enzymes and emulsifiers. Combinations of these are often used to improve the gluten-free product’s rheological characteristics. Some researchers have also invested time and effort in breeding low-gliadin wheat varieties. In general, the recipe alterations for gluten-free bread unfortunately often also lead to an increased product price [15].

Starch naturally occurs in wheat-based products, as 80% of wheat flour consists of starch. Although starch predominantly acts as a quasi-inert filler material during the initial phases of breadmaking, (part of the) starch gelatinizes upon baking. As a result, starch plays a key role in the structure setting of bread. Hence, the elimination of wheat flour also removes starch from the product recipe. Starches of alternative (gluten-free) sources, such as cassava, tapioca, corn, potato, bean, and rice, have been added to gluten-free recipes [16,17,18,19]. In recent years, a gluten-free wheat starch was also developed, and has been tested in combination with rice flour and corn starch for the production of gluten-free products [20,21]. In these studies, it was postulated that wheat starch breads were generally better accepted and had an improved loaf volume compared to the corn starch alternative. However, the gluten-freeness of wheat starch preparations is a controversial theme. Starch naturally displays wide variability in terms of morphology, gelatinization behavior, and viscosity yielding. The importance of starch granule morphology was studied using rice starches. In this study, it was found that round starch granules were preferred over polygonal starch structures for product quality [22]. The underlying reasons for this could, however, expand beyond morphology, and be governed by a rapid gelatinization and good viscosity retention after gelatinization. High viscosity of the batter is essential to build structure in gluten-free food products [22]. In some cases, the natural variability of starch is not sufficient, and starches are then modified to display a specific characteristic. The use of modified starches has also been investigated in the framework of gluten-free products. Acetylated distarch adipate and hydroxypropyl distarch phosphate were found to increase bread loaf volume, produce a more elastic bread crumb, and result in a slight decrease in hardness and chewiness of the bread crumb [23].

In addition to starch, gluten-free flours have also been used as base ingredients (Table 1). Examples are flours of pseudocereals, such as amaranth, buckwheat, chia and quinoa, but also cereal flours that do not contain gluten, such as sorghum, rice, corn, teff, and millet. The use of oat flour is controversial, but previous studies have shown that a moderate consumption of oats does not trigger any adverse health effects in most CD patients [24]. However, the oats that are used need to be certified gluten-free. Oats often become contaminated with gluten-containing cereals during harvest, and the separation of these gluten-containing grains and oats is not that straightforward. Besides cereal and pseudocereal flours, legume (chickpea, pea, carob germ, carob, marama bean, and soy) and chestnut flours have also been successfully used in gluten-free bread applications [25,26,27,28,29,30]. The properties of the used gluten-free flours, such as particle size, starch damage, and fiber content, significantly impact the resulting bread characteristics [31]. This complicates the comparison of the outcomes of different studies on the performance of different ingredients in gluten-free products.

One of the additives often used as a processing aid and/or quality-improving minor ingredient, is dietary fiber (Table 2). The addition of dietary fiber does not only compensate for the nutritional loss of dietary fiber when excluding wheat flour or whole meal from the product recipe, but it also introduces an ingredient with excellent water-binding, viscosity-increasing, and even gel-forming capacities. As a result, product thickening and texturizing characteristics are re-introduced in the gluten-free process. Examples of dietary fiber that were used in gluten-free products are β-glucan, inulin, oligofructose, linseed mucilage, apple pomace, carob fiber, bamboo fiber, polydextrose, and resistant starch [32,33,34,35,36,37]. Fiber structure and molecular weight play a crucial role in gluten-free bread quality [38]. An alternative way of introducing fiber in the (sourdough) bread recipe was explored by Wolter and colleagues [39]: exopolysaccharide (essentially a dextran) production by bacteria, such as *Weissella cibaria*, was found to increase dough strength [39].

Hydrocolloids (Table 3) are essentially polymers that display thickening properties through the binding of water. As a result, the viscosity of the gluten-free “dough/batter” is enhanced and gas is better retained in the “dough” matrix, which increases bread loaf volume and improves loaf crumb structure. The most popular hydrocolloids are xanthan gum and hydroxypropyl methyl cellulose (HPMC) [40,41,42,43,44]. Other gums that have been studied are pectin, guar gum, locust bean gum, agarose, tragacanth gum, cress seed gum, and carboxymethyl cellulose [26,40,45,46,47,48,49].

Gluten is essentially a protein, and it is logical to explore alternative proteins to make up for the loss of gluten functionality in gluten-free products. In the absence of other structure-forming molecules (such as gums), the structure-forming capacity of proteins was explored. Non-gluten proteins have largely varying effects on dough rheology, as well as final bread characteristics and appearance. In the study by Ziobro and colleagues [50], the most promising protein in terms of volume increase was albumin, whilst pea and lupine proteins were preferred over soy protein, sensory-wise. Examples of proteins used are legume, egg, dairy, and non-gluten cereal proteins [13,43,51,52,53,54]. These alternative proteins/protein sources often also display a better amino acid profile than gluten, which is deficient in essential amino acids such as lysine, and hence, cannot be considered as a “balanced” protein. As a result, these alternative proteins are preferred over gluten, from a nutritional perspective. These proteins also lead to a well-appreciated sensory pallet, as they are involved in Maillard browning reactions, which do not only improve product color but also flavor (compared to, e.g., hydrocolloid-based gluten-free products). However, the increased darkness of the bread may not always be perceived as exceptionally desirable.

Enzymes, as processing aids, are often chosen based on their potential to induce the formation of crosslinks in between the polymers present in the product recipe, thus triggering the formation of a network, similar to what would be the case if gluten was present in the recipe. Examples of studied enzymes are transglutaminase, glucose oxidase, tyrosinase, and laccase [55,56,57]. In addition, proteolysis, through the addition of peptidases, has also been explored. These enzymes, similar to what glutathione addition would trigger in bread recipes, lead to depolymerization [58]. In rice flour, for example, it has been shown that the degradation of the high molecular weight protein fraction of rice is needed to allow small protein aggregates to crosslink through disulfide bonds. This process helps to ensure better rheological properties, improved gas retention during baking, and increased overall product quality [59,60]. Starch hydrolyzing enzymes such as alfa-amylase and amyloglycosidase, were added in some product recipes as well [45]. One of the reasons for alfa-amylase addition is the in situ production of sugars to sustain yeast activity [61].

In gluten-free bread products, emulsifiers such as diacetyl tartaric esters of monoglycerides [40], mono- and diacylglycerol [41], lecithin [62] and sodium stearoyl-2-lactylate [63] are used to establish better interactions between the different ingredients. Emulsifiers may also play a role in stabilization of interfaces such as water/air or water/lipid interfaces. The former interfaces are especially important to the fine crumb structure of gluten-free bread loaves. Some proteins are known to display good interface stabilizing properties as well.

#### 2.2.2. Processing

In addition to rational ingredient and/or additive choice, different processing paths have also been explored to alter the gluten content of gluten-containing flours and improve rheological properties of gluten-free products, particularly gluten-free dough.

Gluten-containing flours have been used in combination with protein hydrolysis strategies or sourdough fermentation to produce gluten-free or so-called ‘gluten-reduced’ bread products. Both the aforementioned technologies are believed to eliminate, or at least significantly reduce, gluten levels in dough. Detoxification of gluten through proteolysis targeting proline and glutamine peptide bonds has been explored recently [81]. These proteolytic enzymes essentially cleave those peptide bonds that human peptidases cannot affect. The hydrolysis products should be broken down to less than nine amino acids in order not to trigger any reaction in the gastrointestinal tract of people suffering from CD. Similarly, using sprouted grain to formulate products safe for coeliac patients is based on extensive protein hydrolysis, reducing the immune response to the hydrolyzed gluten in the product. However, in the latter case, sprouting conditions need to be tightly controlled and monitored, not only to ensure proper gluten hydrolysis, but also to retain some wheat flour functionality for baking applications. Sourdough fermentation is another strategy which is believed to reduce the level of immunoresponse-triggering gluten. In this framework, the right selection of lactic acid bacteria that display peptidase activity hydrolyzing the appropriate peptide bonds is crucial [82].

In addition to modifying wheat flour, the gluten-free flours can also be processed in a particular way to change their rheological behavior in dough-like systems. A myriad of different strategies has been explored:

- Corn flour has been milled in various instruments. Different corn varieties were selected to explore the varietal effect and the flour’s physical properties’ impact on its potential to produce high quality gluten-free products [83].
- Germination of brown rice was studied as a pre-treatment to alter the functionality of brown rice flour in gluten-free bread baking applications [84]. Rice germination did indeed alter the hydration and pasting properties of the flour. This resulted in increased crumb softness. However, the germination process had to be closely monitored to control the activity of α-amylase.
- Similar to wheat flour-based systems, sourdough fermentation of teff flour products has also been explored [68,85]. The fermentation was shown to have a major impact on the physicochemical properties of teff starch and a more limited effect on the protein fraction. Bread loaves made with this fermented teff flour yielded better gluten-free breads than those produced with unfermented teff flour [85].
- Phosphorylation of rice flour is another strategy that was studied. The resulting gluten-free breads had a lower hardness and an improved bread volume, crumb appearance, and color [86].
- Pre-gelatinization of the starch used as a base ingredient has also been attempted and led to a decreased dough elasticity, but a higher resistance to deformation, assuring a better retention of gas in the dough structure. As such, hardness of the product was decreased [57].
- Heat treatment has been explored to unlock a specific functionality. Buckwheat grains e.g., have been puffed prior to milling and use in gluten-free bread recipes [70]. Steaming or roasting of soybeans was found to reduce the beany flour of whole soy bread [87].
- Extrusion of rice flour increased the dough consistency and hydration of rice flour gluten-free bread, while increasing the crumb hardness and lowering the specific volume. However, these bread quality effects can be counteracted by working with flours with coarser particle sizes [88].
- Particles of whey protein were shown to display elastic and strain hardening characteristics when mixed with starch. Whey protein has been converted to whey protein particles using a cold gelation method prior to being used to produce gluten-free bread [89]. Van Riemsdijk and colleagues [90] found that the effect of whey protein particles on bread quality was heavily governed by the amount of disulfide bonds present in the dough (and the particles).

## 3. Gluten-Free Diet

The origin of the gluten-free diet dates back to 1941, when it made its debut in a report on the dietary treatment of CD by paediatrician and scientist Willem Karl Dicke [90]. Today, the diet continues to be applied and investigated for a variety of additional health purposes, including the management of NCGS, irritable bowel syndrome (IBS), diabetes, DH, inflammation and obesity.

Research interest in the gluten-free movement, in addition to the clinical and practical uses of the diet, has been growing for many years. In what follows, the current trends, attitudes, and knowledge surrounding the gluten-free diet, as well as its nutritional adequacy, will be covered. Furthermore, the implications of the gluten-free diet on gluten-related conditions, diabetes and other autoimmune diseases, as well as weight management, will be explored.

### 3.1. Consumers’ Motivations, Knowledge and Attitudes

#### 3.1.1. Consumers’ Motivations

Over the past decade, surveys have been conducted to better understand the underlying reasons behind rising trends in gluten-free living [91,92,93,94]. While the clinical diagnosis of CD influences adherence to the gluten-free diet, data indicate that this disease affects less than 1% of the general population [95]. Studies show that adverse symptoms, as well as individual efforts to manage them without a clinical diagnosis, impact gluten-avoidance behavior [92,93]. Symptomatic self-management strategies involving a gluten-free diet have been supported by ethnographic research findings as well [96]. In their field-based study, Copelton and Valle [96] learned that a self-imposed gluten-free diet was common among individuals presenting with unexplained symptoms for extended periods of time. The anticipated length of time, invasiveness, and frustrations associated with diagnostic tests persuade many of these people to eliminate gluten from their diet on their own [96].

Expected health benefits of the gluten-free diet also influence dietary decisions. Although beneficial effects of the diet have yet to be demonstrated in healthy individuals [97,98], consumer market survey data demonstrate that 33% and 26% of Canadians and Americans, respectively, believe gluten-free products are healthier [99,100]. These trends are consistent with the findings of another study investigating health beliefs surrounding the gluten-free diet [101]. In Dunn et al.’s [101] study, 31% of participants believed that gluten avoidance would promote general health, whilst 37% felt that gluten-free products were healthier than their conventional equivalents. Weight loss was reported as another common motivator for adopting the gluten-free diet, especially among younger adult populations [94]. However, evidence supporting the effectiveness of a gluten-free diet in weight management is limited, as discussed later.

#### 3.1.2. Consumers’ Knowledge

Mixed rationales for following the gluten-free diet may be reflective of society’s limited understanding of gluten and gluten-free food formulation. In the United States, survey findings from 1012 respondents suggested that, although gluten-awareness is high, a substantial proportion of citizens can neither describe what it is, nor determine product sources of it [94]. These conclusions have been consistent among smaller studies indicating confusion around gluten-free terms [102], and issues identifying the gluten content of foods [103]. Dietitians also express concern for clients with CD who struggle to identify safe options due to their limited knowledge about gluten-free foods [104].

According to Halmos et al.’s [105] findings, the greatest challenge may lie in identifying gluten-free ingredients rather than gluten-containing ones. This supports the results of an earlier study by Zarkadas et al. [106], in which 85% of respondents with CD struggled to determine whether or not certain foods were gluten-free (*n* = 2681). Lack of knowledge surrounding the diet has implications for both CD patients and the general public. Leffler et al. [107] reported that individuals commonly overestimate their adherence to the diet. Recent literature points to the fact that an inadequate understanding of the diet may not only lead to an unintentional ingestion of gluten, but also to an over-restriction of certain foods and poor adherence to the diet overall [103,105,108].

Research has also explored the most common sources of information on gluten and gluten-free diets. Questionnaire-derived data indicate that popular sources of gluten-free information include the internet, print media sources, cookbooks, coeliac support groups, and other coeliac patients or individuals on the diet [92,103,109,110]. Compared to dietitians, family physicians were found to be less likely referred to for gluten-free information [103], and were rated low or lowest with respect to usefulness [109,110].

#### 3.1.3. Consumers’ Attitudes

Individuals follow the gluten-free diet to varying degrees [101,103]. This may be influenced by the attitudes that people share toward the diet. For example, purchasing gluten-free products may have some negative economic consequences, especially for low-income families [104,109,111]. In contrast to their gluten-containing counterparts, gluten-free products are considerably more expensive. In fact, gluten-free items were reported to be approximately 200–500% more expensive than the equivalent standard products, depending on the product and shopping location [112,113,114,115]. Thus, the affordability and long-term sustainability of the diet continues to spark evaluation.

The inadequate availability of high quality gluten-free items is another burden. Many people report challenges locating such products in local grocery stores [106,111], where their availability varies across shopping venues [113,115]. Individuals with lower socioeconomic status, with limited resources available, or those living in remote cities are certainly at a disadvantage.

Other opinions about the diet focus on the sensory aspects of gluten-free products, as well as the impact it has on many personal and social domains. While consumers may be relatively satisfied with the taste and texture of gluten-free products, continued efforts to improve the palatability of these items are still being urged [111]. Furthermore, individuals avoiding gluten express a lack of confidence when eating outside of the home [106,116], while many find the length of time involved in the at-home preparation of gluten-free options a nuisance [110].

### 3.2. Nutritional Implications

The nutritional adequacy of the gluten-free diet and associated products has always been a concern for consumers, health care professionals, and the industry. While the gluten-free diet is known to alleviate symptoms and promote gastrointestinal healing in patients with gluten-related disorders, long-term adherence to the diet may have concurrent nutritional limitations.

The nutritional profiles of gluten-free products, as well as the dietary intake patterns of individuals on the diet, were assessed in several studies. According to Do Nascimento et al. [117], gluten-free products share a common composition of raw ingredients, including corn, rice, soy, cassava, and potato. These ingredients replace gluten-containing grains like wheat, rye, and barley in regular products. Overall, gluten-free items are higher in fat, sugar, and sodium compared to regular products, though compositional trends may vary by product type [118]. Studies have shown that the total fat content of gluten-free breads is at least twice the amount found in their gluten-containing counterparts, contributing to the improved mouthfeel of these products [119,120]. Conversely, many gluten-free pasta products appear to have significantly higher carbohydrate [120] and sodium contents [121]. Gluten-free products are generally inferior sources of protein and dietary fiber as well [118,119]. The glycemic index (GI) of gluten-free products varies based on the type and quality of ingredients used, as well as the food-processing procedures performed to manufacture them [121]. Since gluten-free items are not typically fortified or enriched in the way that many regular products are, they are also generally lower in folate, iron, niacin, thiamin and riboflavin [122,123]. Efforts have been made to improve the formulation of these products without compromising their sensory appeal [64].

Studies evaluating the dietary intakes of CD patients on a gluten-free diet have reached similar conclusions. According to Barone et al. [124], CD patients, compared to healthy adults, consume significantly higher quantities of fat and sugar, and lower amounts of fiber on the gluten-free diet. Similar findings were reported by other researchers reporting food-record and questionnaire-based data from adults and children [125,126,127,128]. However, it is being questioned whether this trend is reflective of overall dietary habits rather than the gluten-free diet alone [125,129]. For example, while CD patients have been shown to share similar intake patterns of cereal-based products in general with the total population, biscuits and crackers are consumed more frequently among individuals with CD [129]. According to Valitutti et al. [129], the popularity of these high GI products may also reflect consumer dissatisfaction with the palatability and availability of other gluten-free carbohydrate options, such as bread. Finally, inadequate intakes of iron, folate, calcium, selenium, magnesium, zinc, niacin, thiamine and riboflavin, as well as vitamins A and D were reported in CD patients following a gluten-free diet [125,126,127,130,131,132]. While it could be argued that nutrient deficiencies in CD patients can be largely explained by impaired nutrient absorption resulting from CD-associated intestinal damage, Hallert et al. [131] argue that this may not entirely be the case. Despite 10 years on the diet and evidence of mucosal recovery, the total plasma homocysteine levels of CD patients were still higher than average, reflecting ongoing deficiencies in folate, vitamin B6, and vitamin B12 [131].

Dietary evaluations performed on CD patients following a gluten-free diet have largely been based on self or proxy-reported data. As always, it is important to acknowledge the potential for participant bias in these types of studies. Nutritional inadequacies associated with gluten-free diets have been shown to vary by gender and dietary experience [132]. It is therefore, reasonable to assume that gluten-free diet education, health awareness, and other lifestyle factors may influence food choices, and consequently impact study results. Researchers agree that proper follow-up, dietitian collaboration, and nutrition education are important to ensure that those following the diet are not at any additional health risks [120,130,131]. Furthermore, a closer look into fortifying or improving the quality of ingredients in gluten-free products continues to be recommended [121,130].

Media and celebrity endorsements of the gluten-free diet for weight loss have stimulated public interest and driven gluten-free market sales [133]. Empirical evidence confirming the diet’s effects on weight, however, is still unclear. Not only is there limited literature available on the weight-related implications of the diet for the general public, but inconsistent study findings involving CD patient requires further research in this area. To date, the influence of the gluten-free diet on body mass index (BMI), waist circumference, and lipid profiles has been investigated.

It is possible that gluten avoidance might support weight management in healthy individuals, although the evidence is minimal and largely derived from self-reported data [134]. It has been speculated that losses in weight associated with the gluten-free diet may, instead, be a reflection of health-conscious behaviors [134], exaggerated reductions in carbohydrates, and low availability of gluten-free food products [133]. Therefore, contrary to popular belief, there are currently insufficient grounds to verify that gluten elimination results in weight loss for the general public.

Conversely, studies indicate that strong adherence to the gluten-free diet may actually result in weight gain in many CD patients [124,135,136,137,138,139]. For those who are underweight at diagnosis, weight gain on the diet is generally more pronounced and favorable [136,137,138,139]. That said, some evidence suggests that, without adequate dietary counselling, initially overweight and obese CD patients may be at increased long-term health risks on the gluten-free diet [135,136,140]. CD patients may also be more susceptible to developing metabolic syndrome in as early as 1 year on the diet [140]. Nutritional imbalances and shortcomings of gluten-free products, as described in an earlier section, may contribute to some of these changes. On the other hand, a few studies have revealed that a gluten-free diet may help some overweight and obese CD patients lose weight [137,138,139]. Earlier diagnosis, perceived mastery of the diet [139] and counselling by a dietitian [138] were shown to influence positive weight outcomes on the diet.

Variations between studies may reflect regional or cultural differences in the type and quality of foods consumed on the gluten-free diet, as well as individual exercise practices [136,138]. To gain a better understanding of the actual effects of gluten-free diet on weight and weight management, consistent efforts should be made across studies to monitor the dietary and physical activity habits of participants, as well as their compliance to the gluten-free diet. Additional research is recommended to ensure that healthy and CD individuals are appropriately informed and advised.

## 4. Gluten-Related Disorders

**Figure 1:**
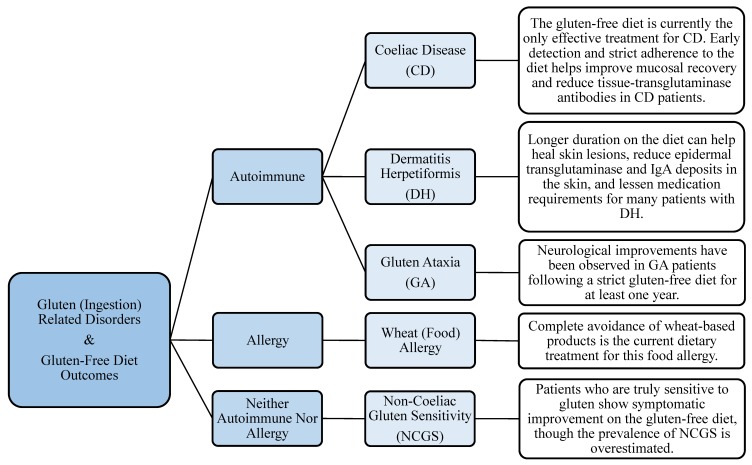
Summary of the effects of the gluten-free diet on the outcomes of gluten-related disorders.

As previously stated, the gluten-free diet is to date, the only effective treatment to a number of gluten-related disorders, including CD, NCGS, GA, and DH. The role of this diet in alleviating the symptoms of these disorders, in part through the modulation of gut microflora, is discussed in the following sections and summarized in Figure 1.

### 4.1. CD, NCGS, GA, and DH

The introduction of wheat to the human diet prompted a myriad of health conditions derived from the body’s immune response to gluten [141]. While there is an overlap in their symptomatic presentation, experts have agreed on several distinctions between these gluten-related disorders [141,142]. Wheat can trigger immunologic reactions depending on its route of exposure, by ingestion, inhalation, or skin contact [141]. When consumed, wheat can act as a food allergen, initiating immunoglobulin E (IgE) or non-IgE mediated reactions [143,144].

CD, DH, and GA are gluten-related autoimmune conditions [141]. CD is a chronic enteropathy involving a gliadin-specific T-cell response, causing inflammation, villous atrophy, and malabsorption in the small bowel of genetically vulnerable individuals [141,145]. In fact, an association between CD and other gastrointestinal and extraintestinal disorders was reported. DH is a common comorbidity of CD and is often called “coeliac disease of the skin” [141,145,146]. It presents as an itchy, blistering rash and is detected by the existence of IgA epidermal transglutaminase antibody complexes in the papillary dermis [147]. Finally, GA is a condition in which cerebellar damage results from the production of antibodies, following gluten ingestion by susceptible patients [141]. NCGS, on the other hand, is diagnosed by exclusion criteria; when a reaction to gluten is evident after both a wheat allergy and CD have been ruled out [142,143]. A significant proportion of patients with IBS have a sensitivity to gluten [148]. Literature indicates that similarities in the clinical presentation of NCGS and IBS generate confusion when evaluating the causes and management options for the manifested symptoms [142,149,150].

#### 4.1.1. Management of Symptoms

One way the gluten-free diet can be beneficial to gluten-related disorders is through the management of related symptoms. Researchers agree that strict adherence to the gluten-free diet offers the greatest relief of symptoms for most patients with CD [151,152,153,154,155]. However, symptom recovery rates differ across age and gender [151,152,153,154]. It is also worth noting that diagnostic delays over 5 years may worsen recovery rates on a gluten-free diet [151].

Changes in CD-related serum antibody concentrations and mucosal recovery rates, following the gluten-free diet, have also been investigated. Studies provide clear evidence of a decline in tissue-transglutaminase antibodies in CD patients on the diet [154,156], even as early as 3 months following a diagnosis [157]. Rates of histological normalization on the gluten-free diet are less consistent across studies [158,159,160,161,162]. Longer duration on the diet, however, appears to improve villous recovery [160]. Adherence to the gluten-free diet, education level, and gluten-free knowledge, as well as age at CD diagnosis, were all shown to influence mucosal recovery [158,160,161,163].

Although results may take months to years, patients with DH have shown significant improvements and better long-term management of symptoms on a strict gluten-free diet. The diet may also provide a protective effect against the development of lymphoma, which is a potential risk for patients with DH and CD [164]. According to the literature, the diet can not only help clear skin lesions, but may also heal the small bowel mucosa, decrease IgA and epidermal transglutaminase deposits in the skin, and reduce the need for oral medications in these patients [165,166,167,168].

Case reports [169,170] and other studies [171,172] indicate that patients with GA may show clinical improvements on a gluten-free diet as well. Neurological benefits, including improved cerebellar function and stabilization of the condition, are influenced by patient adherence to the gluten-free diet [172]. At least one year on the diet may be required for clear signs of improvement to be detected in GA patients [173].

The gluten-free diet’s effectiveness in managing NCGS and IBS symptoms is a popular area of debate. For individuals with NCGS, whose symptoms are provoked by gluten, the gluten-free diet is shown to keep the number and severity of their symptoms at bay [148,173,174,175]. However, randomized, double-blind placebo-controlled challenge studies revealed that the true proportion of gluten-sensitive individuals may be overestimated [174,176,177]. Furthermore, although the gluten-free diet was found to provide some relief for patients with diarrhea-dominant IBS [178,179], the roles of α-amylase/trypsin inhibitors and fermented oligosaccharides, disaccharides, monosaccharides, and polyols (FODMAPs) in triggering IBS-type symptoms cannot be ignored [180,181,182].

#### 4.1.2. Management of Gut Microflora

Another way the gluten-free diet may benefit some gluten-related disorders is through modulation of gut microflora. The gluten-free diet has been shown to beneficially alter the gut bacterial composition and function in individuals with CD [183,184]. The low polysaccharide content of the gluten-free diet could help explain some of the changes observed in the microbiota [185]. Intestinal healing on the diet may also help support the growth of different bacterial species [183]. However, findings are still inconsistent. In adults with CD, Nistal et al. [183] found that adult patients on the diet began to show some changes similar to the microbial community patterns of healthy adults, but that differences in the richness and presence of unknown bacterial communities still existed. In child CD patients, more than 1 year on the diet seems to be needed to restore normal functions of the gut microflora [184]. Differences in the reported benefits also exist between the gut microbial composition of symptomatic and asymptomatic CD patients on a gluten-free diet [186].

Additional studies are still needed for a consensus to be reached. Research of this kind is limited and mostly restricted to small sample sizes. There is also inconsistency in the target age groups across studies, as well as the duration and adequacy of gluten-free dietary adherence. The effects of confounding genetic and other environmental factors on the gut microbiome must also be considered.

### 4.2. Other Disorders Closely Linked to CD

An association was described between type 1 diabetes (T1D) and CD, with a 1–16% prevalence of CD found in T1D cases [187]. In addition to T1D, patients with CD are more susceptible to developing autoimmune diseases including autoimmune thyroiditis, psoriasis, rheumatoid arthritis, Sjögren’s syndrome, DH, and Addison’s disease [188,189].

#### 4.2.1. T1D

Both genetic and environmental elements are known to influence the development of T1D, and researchers suspect that dietary gluten may be one contributing factor [190]. Human studies have produced less conclusive results than pre-clinical studies and additional research is still needed. Variations in the clinical backgrounds, age groups, and dietary compliance of subjects, as well as the lack of controls and small sample sizes across studies might explain the inconsistencies. Owing to the association between T1D and CD, researchers have also investigated the gluten-free diet’s impact on glycemic control in patients with both autoimmune diseases. The gluten-free diet reduced severe hypoglycemia in children with T1D and CD, over the short term [191]. Although the level of clinical significance varies between studies, HbA1c levels were also found to improve in children with T1D and CD following a gluten-free diet intervention [192,193]. However, other studies found no significant improvement in metabolic control in T1D patients with CD following a gluten-free diet [194,195,196]. Furthermore, the high GI of many gluten-free products could put ill-informed T1D patients at risk of a loss of glycemic control [197].

#### 4.2.2. Other Autoimmune Diseases

The role of the gluten-free diet in reducing the risk of comorbidities of autoimmune diseases in CD patients remains unclear. In 1999, Ventura et al. proposed that the incidence of other autoimmune diseases in patients with CD may be linked to prolonged exposure to gluten. Since then, other studies have been conducted to further explore the association between gluten exposure in CD patients and the development of these disorders [188,198,199,200]. Three of the studies revealed that the risk for developing other autoimmune diseases does not appear to be significantly impacted by the duration of gluten exposure in CD patients [188,198,199]. Cosnes et al. [200], on the other hand, reported a possible protective effect of the gluten-free diet. As many of these studies are based on retrospective data [198,199,200], prospective research in this area is advised.

## 5. Conclusions

The replacement of gluten as a vital ingredient in numerous food products is not straightforward. Different ingredients and processing techniques have been investigated to date. However, the quality of gluten-free products is often not comparable to gluten-containing products. More effort should be devoted to a more rational approach which uses the gluten-containing product as the golden standard.

The motivation to adopt a gluten-free lifestyle goes beyond its original application for CD management. Perceived health benefits and relief of adverse symptoms on the diet influence individual decisions to abstain from gluten. Even so, confusion surrounding gluten and gluten-free options, as well as the high cost and low availability of gluten-free products, can be burdensome for many people. For others, drawbacks of the gluten-free diet may be small in comparison to the clinical improvements made on the diet. Despite media claims, there is also limited evidence confirming the diet’s effectiveness in weight loss for the general public. Furthermore, the reported weight gain in CD patients on the diet may not always be favorable, particularly among previously overweight and obese individuals. However, to date, no beneficial effects from a gluten-free diet have been shown in healthy individuals. Most importantly, individuals choosing to follow a gluten-free diet should take caution of the macronutrient and micronutrient inadequacies of the diet. Overall, it is generally recommended that dietary education and counselling be offered to support gluten-free dieters.

## Figures and Tables

**Table 1:** Alternative flours used in gluten-free product formulations with the main quality effects.

Formulation	Main Conclusions	References
Base of corn starch with *chickpea and tiger nut flour*	Replacement of emulsifier and shortening by the chickpea protein and tiger nut lipids: the combination of both maintains baking characteristics of bread loaves with eliminated shortening and emulsifier.	[15]
Base of rice flour, potato, tapioca and cassava starch and xanthan gum with *amaranth and quinoa flour*	Amaranth and quinoa flour do not affect texture and volume, and final bread loaves are considered ‘moderately acceptable’ in sensory trials.	[64,65]
Base of rice flour and xanthan gum with *amaranth, quinoa and buckwheat flour*	Replacement of potato starch with buckwheat and quinoa flour increases bread volume and softens crumb. Amaranth flour only decreases the crumb firmness. None of the three pseudocereal flours adversely affects the sensory properties.	[66]
Base of corn starch with *vinal seed and corn flour*	Acceptable bread loaves are made with regard to volume and crumb structure.	[67]
Base of rice flour, maize starch and HPMC with *teff flour and dried rice- and buckwheat based sourdough*	Bread aroma is enhanced and visual appearance is good. Buckwheat-based sourdough has a bitter taste.	[68]
*Soy flour-barnyard millet* blends	Soy flour alters the textural properties and color of the bread.	[69]
Base of rice flour, shortening, gum blend (xanthan, guar and locust bean gum) and DATEM with partial replacement of the rice flour with *chestnut flour*	Partial replacement of rice flour with chestnut flour results in lower hardness, increased specific volume, and better color and sensory properties. High chestnut flour recipes had low quality.	[40]
Base of rice and corn flour, corn starch, HPMC with gradual replacement of rice/corn flour by *quinoa flour*	Quinoa flour increases loaf volume and yields a more homogeneous crumb structure, whilst not affecting product taste.	[61]
Base of *oats, rice, buckwheat, maize, quinoa, sorghum and teff flour*	Only oats bread is somewhat comparable to wheat bread. All other loaves are of inferior quality in terms of loaf volume, physical crumb texture, shelf life and aroma profile.	[24]
Base of commercial gluten-free mixtures including corn starch, psyllium fiber, guar gum or corn starch, tapioca starch, potato starch and rice flour, HPMC with partial replacement of the flours by *buckwheat flour*	Dehulled buckwheat flour improved the baking performance of commercial mixtures, whilst puffed buckwheat flour had a clear effect on water availability and the interaction between the matrix biopolymers.	[70]
Base of corn starch and xanthan gum with *soy and chickpea flour, pea isolate and carob germ flour*	Carob germ flour loaves have the lowest volume, whilst chickpea flour yields the highest volume and the softest crumb.	[25]
Base of *marama bean flour* with cassava starch	Marama bean and cassava starch produce strong dough, similar to wheat flour dough that can hold gas in its structure.	[27]
Base of potato starch and rice flour with *whole chia flour*	Chia flour does not adversely affect loaf volume and crumb firmness.	[71]
Base of rice flour, gluten-free wheat starch, albumin, HPMC with *green plantain flour*	Green plantain flour produces good volume bread loaves, and soft crumb firmness breads having a regular porosity.	[21]
Base of rice flour and corn starch with *acorn meal*	Sensory and nutritional properties are improved with acorn supplementation, whilst the specific volume is decreased, and the crumb hardness is increased.	[72]
Base of corn starch, HPMC with *carob germ flour*	Carob germ flour is a good alternative to wheat flour to produce viscoelastic dough and high quality gluten-free bread.	[73]

DATEM, Diacetyl Tartaric Esters of Monoglycerides; HPMC, hydroxy propyl methyl cellulose.

**Table 2:** Hydrocolloids used in gluten-free product formulations with the main quality effects.

Formulation	Main Conclusions	References
Zein-starch base with *HPMC* and high β-glucan oat bran	Hydrocolloid and β-glucan improve bread volume and aid zein to more closely resemble gluten in terms of structural and rheological properties.	[74]
Base of soybean flour and corn starch with *HPMC, xanthan gum* and emulsifiers	HPMC increases volume and softness more than xanthan gum, but xanthan gum gives a better crumb structure.	[41]
Base of teff, buckwheat, corn or rice flour with *HPMC and xanthan gum (combinations)*	Xanthan gum increases the crumb hardness of teff and buckwheat breads, whilst corn breads become softer. HPMC increases loaf volume of teff and corn breads, while xanthan adversely affects the loaf volumes in all different recipes.	[42]
Base of rice flour, corn starch and sodium caseinate with *pectin, carboxymethyl cellulose, agarose, xanthan gum* and oats β-glucan	Except for xanthan, all gums result in a loaf volume increase.	[46]
Base of potato flour with *HPMC, carboxymethyl cellulose, xanthan gum and apple pectin*	Gums yield loaves with higher specific volume and reduced hardness.	[47]
Base of rice flour, corn starch, soy flour with *guar gum* and transglutaminase	Guar gum increases the specific volume and decreases crumb hardness, while transglutaminase increases crumb hardness but yields a good texture.	[56]
Base of chestnut and chia flour with *guar gum, HPMC and tragacanth gum*	All hydrocolloids increase “dough” elasticity.	[26]
Base of rice flour, corn starch and sodium caseinate with *carboxymethyl cellulose*	Carboxymethyl cellulose increases bread volume and sensorial properties.	[48]
Base of broken rice berry flour with *guar, locust bean or xanthan gum*	Hydrocolloids increase loaf volume, texture, microstructure and sensory properties.	[75]
Base of tapioca starch, precooked corn flour with *guar gum and HPMC*	Guar gum and HPMC reduce dough stickiness and soften the crumb.	[76]
Base of rice and corn flour and corn starch with *cress seed and xanthan gum*	Both gums improve crumb color and porosity, cress seed gum triggers the formation of more regular and solid pores.	[51]

HPMC, hydroxy propyl methyl cellulose.

**Table 3:** Fiber (sources) used in gluten-free product formulations with the main quality effects.

Formulation	Main Conclusions	References
Base of corn flour, corn starch, dried eggs and carrageenan with *psyllium and pea fiber and oat bran* and glucose oxidase	Addition of dietary fiber alters dough cohesion and starch pasting properties. (Glucose oxidase increased the specific loaf volume).	[77]
Base of corn starch, rice flour, starch and protein, HPMC, locust bean gum, guar gum and alfa-amylase with *psyllium and sugar beet fiber*	Both psyllium and sugar beet fiber improve dough workability. Psyllium fiber is superior in its film forming ability and has an antistaling effect due to higher water binding capacity.	[45]
Base of rice and corn flour, corn starch, HPMC with *quinoa bran or quinoa wholemeal addition*	Quinoa bran increases carbon dioxide production, while the gas retention is reduced. Bread volume can be increased without adversely affecting the taste.	[78]
Base of corn and potato starch, pectin, guar gum with replacement of pectin and guar gum with *linseed mucilage (predominantly arabino-xylan)*	Replacement of pectin or guar gum with linseed mucilage improves the sensory acceptance and does not affect texture and bread staling.	[32]
Base of rice flour, corn starch and HPMC with *insoluble fiber (oat and bamboo, pea and potato fiber) and soluble fiber (barley and polydextrose)*	Soluble fiber decreases dough consistency, increases bread volume and decreases crumb hardness. The fine insoluble fibers also increase bread volume and decrease the crumb hardness, the coarse insoluble fibers decrease bread volume and increase hardness. In general, soluble fiber increases the structural stability, while insoluble fiber disrupts the structure.	[33]
Base of rice flour, HPMC with *β-glucan derived from barley (low molecular weight) and oats (high molecular weight)*	Low molecular weight β-glucan develops a gel network structure, whilst high molecular weight β-glucan predominantly increases viscosity.	[79]
Base of white rice, corn and buckwheat flour with *carob fiber*	Carob fiber improves volume, color and crumb texture whilst increasing the antioxidant activity of the breads.	[36]
Base of rice flour, cassava starch, full-fat active soy flour with *inulin (soluble fiber) and resistant starch and oat bran (insoluble fiber)*	Insoluble fiber increases dough firmness and decreases loaf volume, whilst soluble fiber decreases dough firmness.	[37]
Base of corn and potato starch, guar gum and pectin with *inulin*	Inulin addition leads to an increased loaf volume and reduces crumb hardness, whilst the internal structure is more polydisperse.	[80]

HPMC, hydroxy propyl methyl cellulose.
